# Combined analysis of gene regulatory network and SNV information enhances identification of potential gene markers in mouse knockout studies with small number of samples

**DOI:** 10.1186/1755-8794-8-S2-S10

**Published:** 2015-05-29

**Authors:** Benjamin Hur, Heejoon Chae, Sun Kim

**Affiliations:** 1 Interdisciplinary Program in Bioinformatics, Seoul National University, Daehak-dong, 151-742 Seoul, Korea; 2 Department of Computer Science, School of Informatics and Computing, Indiana University, 150 S. Woodlawn Avenue, 47404 Bloomington, USA; 3 Department of Computer Science and Engineering, Seoul National University, Daehak-dong, 151-742 Seoul, Korea

**Keywords:** Gene marker, Differential expressed genes, Gene regulatory network, Knockout mice, Single-nucleotide variants, Small number of samples

## Abstract

RNA-sequencing is widely used to measure gene expression level at the whole genome level. Comparing expression data from control and case studies provides good insight on potential gene markers for phenotypes. However, discovering gene markers that represent phenotypic differences in a small number of samples remains a challenging task, since finding gene markers using standard differential expressed gene methods produces too many candidate genes and the number of candidates varies at different threshold values. In addition, in a small number of samples, the statistical power is too low to discriminate whether gene expressions were altered by genetic differences or not. In this study, to address this challenge, we purpose a four-step filtering method that predicts gene markers from RNA-sequencing data of mouse knockout studies by utilizing a gene regulatory network constructed from omics data in the public domain, biological knowledge from curated pathways, and information of single-nucleotide variants. Our prediction method was not only able to reduce the number of candidate genes than the differentialy expressed gene-only filtered method, but also successfully predicted significant genes that were reported in research findings of the data contributors.

## Background

RNA-sequencing (RNA-seq) data are widely used for detecting differential expressed genes (DEGs) [1,2] and DEGs are often used for finding gene markers to explain the phenotypic differences between control and cases. However, in gene knockout studies, discovering gene markers with the DEG-based method has several limitations. It is known to be difficult to distinguish whether the expression alteration resulted from the inactivation of the knockout gene that caused phenotypic differences or from the genetic variations that were merely from differences in samples rather than phenotypic differences. The problem of distinguishing gene markers from the DEG-based method becomes much more challenging when the number of samples is small [3], an issue that RNA-seq experiments face frequently.

Various methods and models were proposed to overcome the difficulties of selecting phenotype related DEGs from a small number of samples such as the Poisson model [4], Bayesian approaches [5,6], and increasing the sequence depth of samples [3]. Even though the intensive studies have resolved the difficulties of DEG detection in some degree, there is no method that can effectively suggest phenotype related DEGs from a small number of samples. In order to obtain significant DEGs, a recent study suggests increasing the number of biological samples rather than increasing the sequence depth [7]. However, increasing the number of biological samples is not easy for many reasons including budget issues, hence a new approach that can detect significant gene markers in a small number of samples is neccesary.

In this study, we propose a new method that distinguishes gene markers with a small number of samples from case-control studies (especially, in knockout studies), by combining multiple methods such as DEG, gene regulatory network (GRN), biological pathways and single-nucleotide variants (SNVs), in a single computational framework.

## Proposed method for selecting phenotype specific DEGs

**Figure 1:**
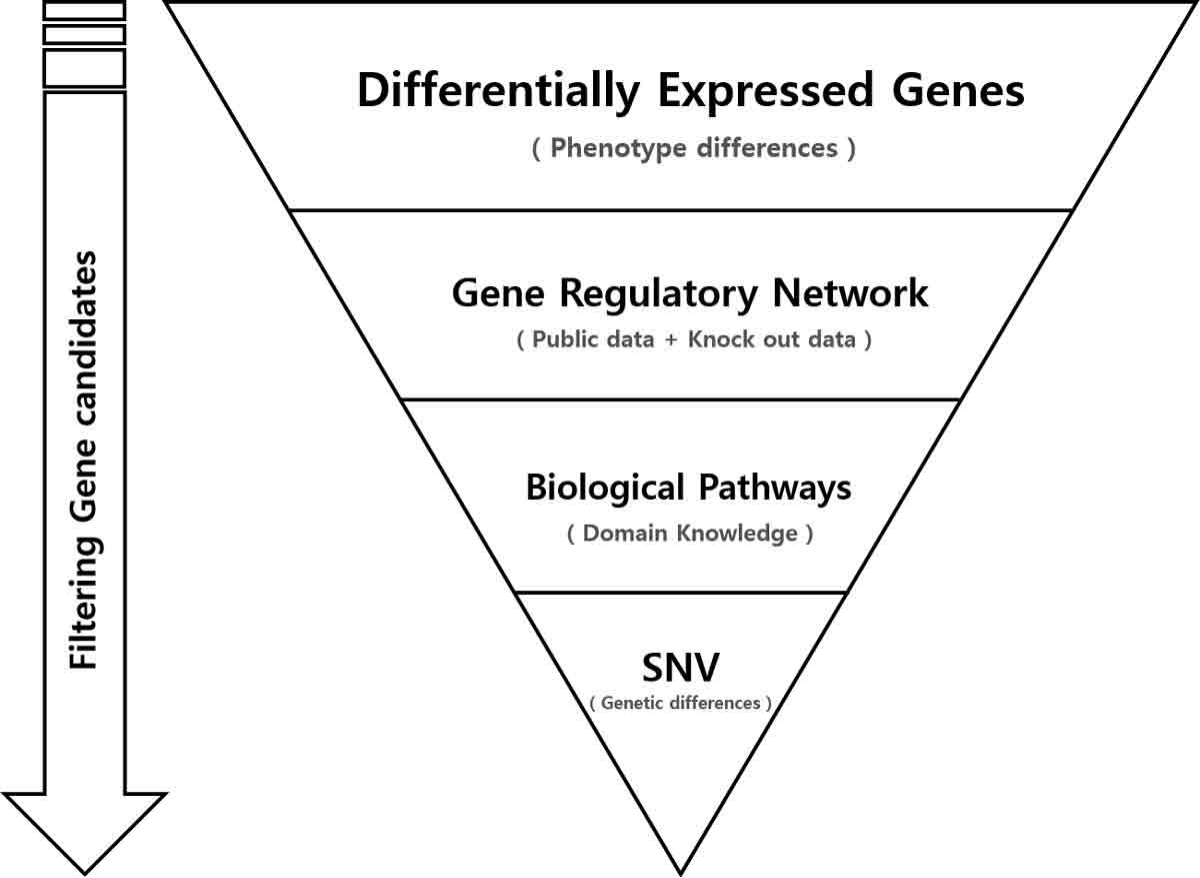
**Overview of the gene marker selection method**.

Our gene marker selection method is a reductionist-approach by adding more filters at each step as described below. The order of filtering steps is illustrated in Figure 1.

1. The first filter is to use a method to identify DEGs between control and case samples. In this study, we used fold change, a classical DEG selection method.

2. The filter at the second step is to use GRN. GRN is constructed from large volume of public data to represent the whole gene regulatory network. DEGs that are included in the network are selected as candidates.

3. The third filter utilizes biological pathway information. Candidates that are not included in the pathways are discarded.

4. Finally, candidates that have higher than a certain rate of SNVs are discarded since the DEGs that have SNVs possibly resulted from genetic difference rather than phenotypic differences.

### Candidate selection using DEGs

In the first step, DEGs were chosen as initial candidates of gene markers. DEGs are used for the purpose of observing the alteration of expression patterns that could explain the phenotypic differences among samples. DEGs were selected by using fold change of the expression value (FPKM) between case and control. We used multiple cutoffs in order to compare and observe differences in the number of selected genes. In addition to candidate selection using DEGs, conditions such as up regulation and down regulation were recorded at each threshold. As shown in Table 1, the numbers of DEGs decreases significantly when the cutoff value increases. However, it is difficult to determine which cutoff is appropriate to be considered as candidates. In addition, the number of candidates are still too big even at the most restricted threshold. Moreover, it is not clear whether the expression alteration resulted from the differences of phenotype or not. Therefore, the necessity of seeking additional information arises, and needs to be combined to the initial candidates in order to select more significant DEGs that can explain whether the expression alterations resulted from the phenotypic differences or from the genetic differences.

**Table 1:** Number of genes selected by each method.

Feature	DEG	DEG+GRN	DEG+GRN+Pathway	DEG+GRN+Pathway+SNV
**Fold change (KO / WT)**	**Number of candidate genes**	**Number of candidate genes**	**Number of candidate genes**	**Number of candidate genes**

More than 1, Less than 1	12298	8834	3436	2622
More than 1.1, Less than 0.91	8953	6264	2441	1861
More than 1.2, Less than 0.83	6466	4322	1683	1272
More than 1.3, Less than 0.77	4631	2986	1179	879
More than 1.4, Less than 0.71	3495	2125	845	629
More than 1.5, Less than 0.67	2712	1574	633	463
More than 1.6, Less than 0.63	2153	1184	478	343
More than 1.7, Less than 0.59	1750	914	364	257
More than 1.8, Less than 0.56	1439	718	284	203
More than 1.9, Less than 0.53	1235	562	233	165
More than 2.0, Less than 0.5	1064	456	192	135

### Reducing candidates by combining GRN

In order to select significant gene markers from the candidates selected by DEGs, we need to investigate which candidates are involved in the regulatory roles that caused phenotypic differences by comparing wild type (WT) and knockout (KO) mice. Therefore, GRN is used for the purpose of discarding non-significant genes, as GRN is a very effective method that can consider complex relationships of many genes [8] (in here, candidate genes). NARROMI [9] was used for GRN construction. Details of GRN construction are described in Methods section. Using the topology information created by NARROMI, we discarded those candidates that have weak or no regulatory role. In other words, potential regulatory roles should exist between two DEGs. As a result, genes that have regulatory roles remained as candidates. Table 1 shows the result of a reduced number of candidates.

### Reducing candidates by combining biological pathways

The combination of DEG and GRN information was used not only for reducing the number of candidates but also to select significant genes that have regulatory roles that could represent the phenotypic differences between WT and KO mice. However, it is also important to ensure whether the candidates are related to the curated biological pathways or not. Even though the candidates were selected by considering their regulatory roles from the gene complex, it is still not clear whether they really have a role in pathways. Therefore, confirming the candidates in terms of domain knowledge is necessary. In this study, we used KEGG pathway [10] for the confirmation. Candidates that do not have corresponding information to the KEGG pathway are excluded from the candidates. Table 1 shows that use of biological pathways was able to narrow down the candidates significantly.

### Reducing candidates by combining SNV information

Even though we reduced the number of candidates by using multiple filtering methods, it is necessary to eliminate genes that have genetic differences that may not represent phenotypic differences. However, since the statistical power is weak in a small number of samples, it is difficult to distinguish whether the genetic differences were caused by phenotypic differences or not. Therefore we used a simple naive solution that removes genes that have certain or higher SNV rate. This is to avoid the risk of selecting SNVs resulting from genetic differences. However, this process will remove SNVs that caused not only non-phenotypic differences but also phenotypic differences. Details of candidate reduction using SNVs are explained in the Methods section.

## Results and discussion

**Figure 2:**
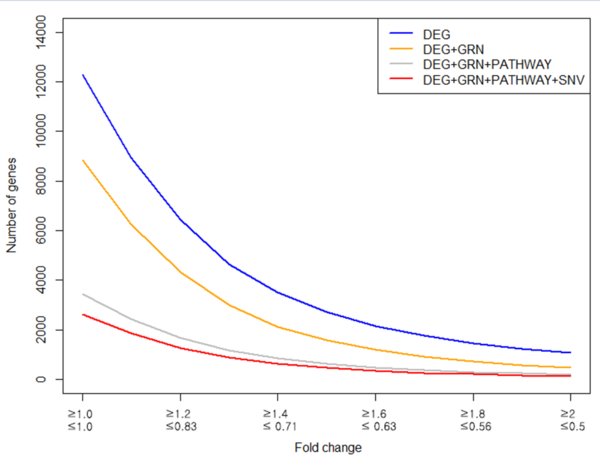
**Comparison of the number of candidates of each method at each fold change**.

To show the effectiveness of our proposed method, we used RNA-seq data of GSE47851 from NCBI Gene Expression Omnibus (GEO) [11]. GSE47851 is from a study that used Gata3 conditional deficient mice [12]. Specific details of preprocessing of RNA-seq data are described in the Methods section. We used DEG, GRN, biological pathways and SNV information to the RNA-seq data in order to find gene markers that can distinguish the phenotypic difference between WT and KO. The gradual combination of multiple filters showed a dramatic reduction of the number of genes in each step (Table 1, Figure 2). Since it is known that defining DEG by fold change is an arbitrary selection, it is necessary to compare the differences at each threshold. We used various fold changes in order to observe the differences in the number of candidates at each threshold. However, we were intrigued by the results that the number of candidates varies lesser in combined filters (DEG, GRN, pathway and SNV) than other filtering methods. Figure 2 interprets that the number of candidates in fold change over 1.6 and less than 0.63 varies less than other threshholds. Therefore, we decided to use fold change 1.6 and 0.63 in order to validate our study.

The biological significance of gene markers that were selected by our method is discussed in terms of the research article that used the GSE47851 data set. Selection of gene markers was done entirely by computational analysis without resorting to the literature and our prediction results were compared with findings of the data contributor. It is reported that genes of TNF and TNFR super families, members of NFkB and cellsurface markers of ILC2s have expression alterations when Gata3 is not activated in ILC2 cells [12]. The following study stated:

"we found that many TNF and TNFR superfamily genes, such as **Tnfrsf9 **and T̶n̶f̶s̶f̶2̶1̶ and NFkB family members, including **Nfkb2 **and **Relb**, showed altered expression patterns. In addition, the cell-cycle inhibitor **Cdkn2b **was up regulated upon Gata3 inactivation".

**Figure 3:**
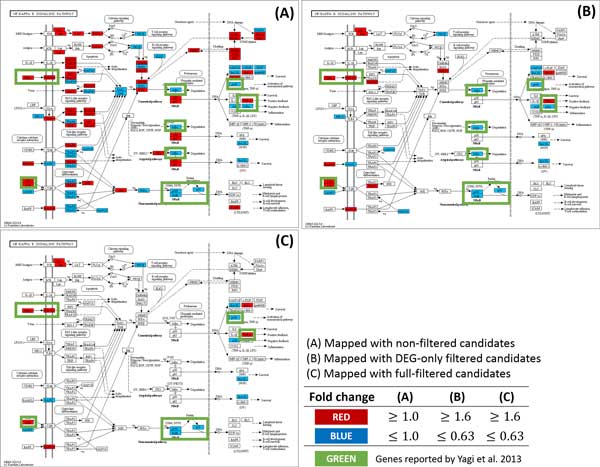
**Comparison of the number of the gene marker candidates between non-filtered and filtered method in NF-kappa B signaling pathway**. (A) NF-kappa B signaling pathway mapped with non-filtered candidates. With no filtering method, too many genes are shown in the pathway which makes it difficult to find an appropriate gene marker. (B) NF-kappa B signaling pathway mapped with candidates filtered by DEG. The number of genes is greatly reduced compared to the non-filtered method. However, difficulty exists in finding significant gene marker as the number of genes are still too many. (C) NF-kappa B signaling pathway mapped with full-filtered candidates. The number of genes was greatly reduced compared to the non-filtered or DEG-only filtered candidates while keeping the genes reported by Yagi et al.(2013).

**Figure 4:**
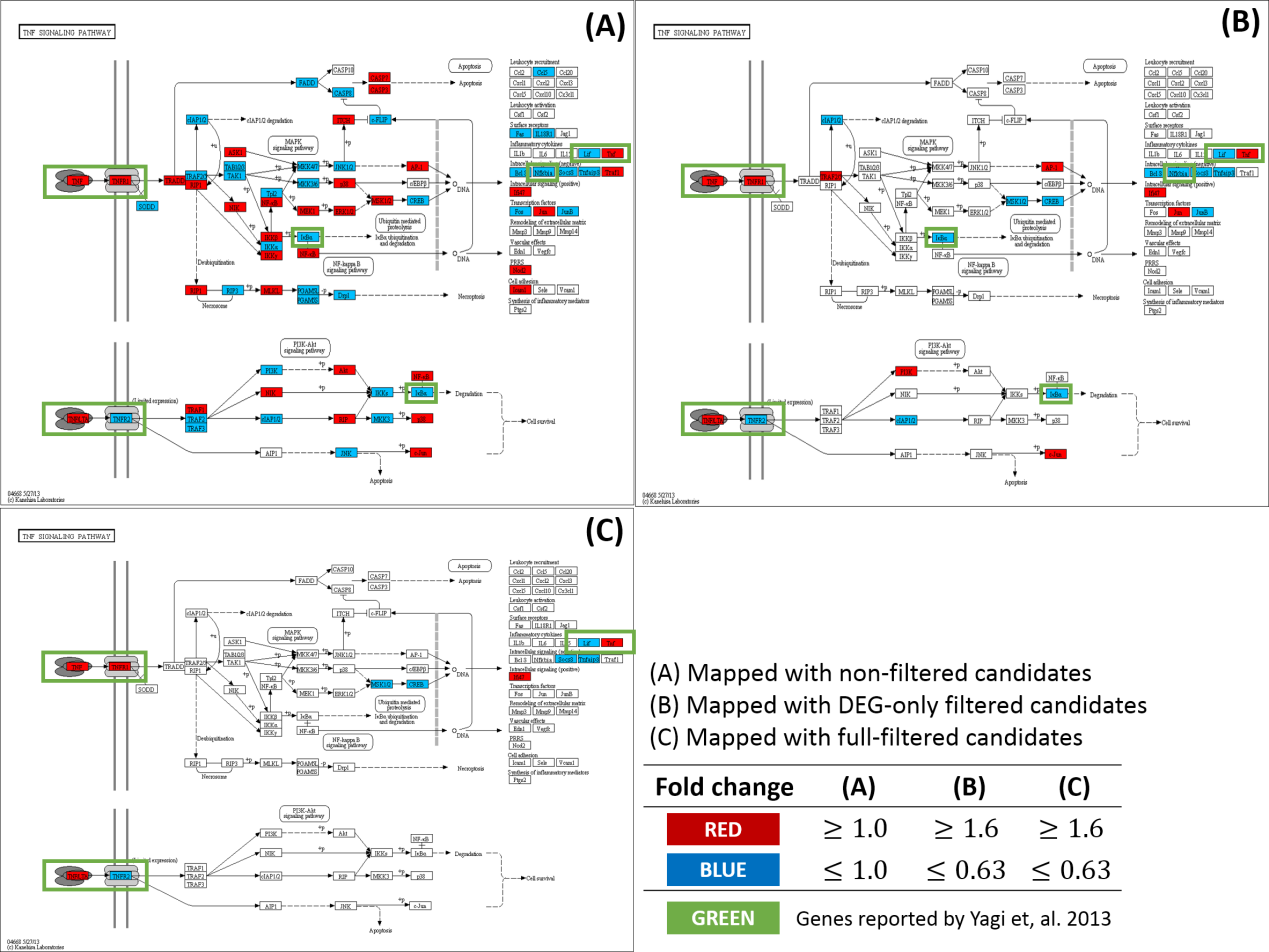
**Comparison of the number of the gene marker candidates between non-filtered and full-filtered method in TNF signaling pathway**. (A) TNF signaling pathway mapped with non-filtered candidates. With no filtering method, too many genes are shown in the pathway which makes it difficult to find an appropriate gene marker. (B) TNF signaling pathway mapped with candidates filtered by DEG. The number of genes is greatly reduced compared to the non-filtered method. However, difficulty exists in finding significant gene marker as the number of genes are still too many. (C) TNF signaling pathway mapped with fullfiltered candidates. The number of genes was greatly reduced compared to non-filtered or DEG-only filtered candidates while keeping the genes reported by Yagi et al.(2013).

**Figure 5:**
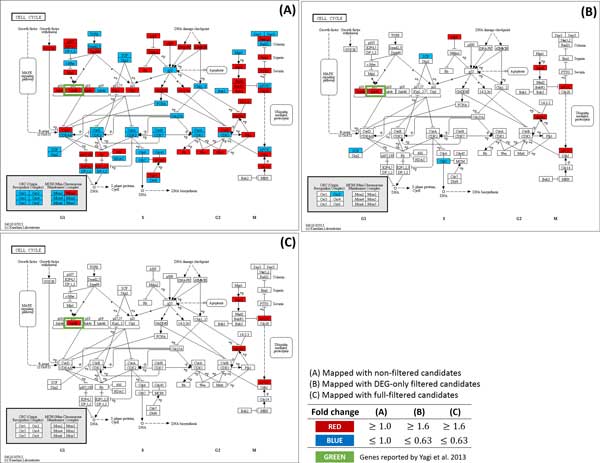
**Comparison of the number of the gene marker candidates between non-filtered and full-filtered method in Cell cycle pathway**. (A) Cell cycle pathway mapped with non-filtered candidates. With no filtering method, too many genes are shown in the pathway which makes it difficult to find an appropriate gene marker. (B) Cell cycle pathway mapped with candidates filtered by DEG. Number of genes are greatly reduced than non-filtered method. However, difficulty exists in finding significant gene markers as the number of genes is still too great. (C) Cell cycle pathway mapped with full-filtered candidates. The number of genes was greatly reduced compared to non-filtered or DEG-only filtered candidates while keeping the genes reported by Yagi et al.(2013).

Our method found 4 out of 5 genes included in the statement above. Where 4 genes are in bold face and un-matched genes are struckthrough. In addtion, we were able to reconfirm the following facts by mapping the candidate genes to the KEGG pathway. Figure 3C represents expression alteration in NF-kappa B signaling pathway, showing down regulations of Nfkb2 (p100) and Relb when Gata3 is inactivated. Expression alteration was also detected in the TNF signaling pathway (Figure 4C). TNF and TNFR super family genes, such as Tnf and Tnfrsf9, were successfully detected in the pathway as well as the statement. In addition, up regulation of Cdkn2b showed in Cell cycle pathway (Figure 5C) which was also stated exactly from the study.

The study also stated about the expression alterations in cell-surface markers of ILC2s:

"130 genes are positively regulated by GATA3 in ILC2s, but not in Th2 cells; they include **Icos**, **Il2ra**, **Kit**, **Il1r2**, C̶y̶s̶l̶t̶r̶1̶, H̶t̶r̶1̶b̶, and T̶p̶h̶1̶, many of which are cell-surface markers of ILC2s."

In the statement above, 4 genes among 7 were successfully matched. Inferring that our proposed method was able to find that the candidates filtered by multiple information were indirectly verified in terms of findings described in the studies that used the data, which shows the effectiveness of determining important genes as potential gene markers.

In addition to the effectiveness, we noticed that our filtering method was able to reduce false positive effectively and helps to focus on significant genes by using biological pathways. Figure 3, Figure 4 and Figure 5 show not only a dramatic reduction in gene numbers in the pathway (from non-filtered to full-filtered) but also candidate genes are likely to be as gene markers. This implies that our proposed method might help researchers focus on genes that may have caused phenotypic differences, hence able to discover true gene markers more efficiently.

We summarize the performance of our method in terms of the gene marker selection. We defined the reported genes (26 in total) [12] as gene markers (true positive) and used them to evaluate of our filter method in terms of precision, recall, F-measure, and accuracy.

(1) $$Precision=\frac{tp}{tp+fp}$$

(2) $$Recall=\frac{tp}{tp+fn}$$

(3) $$F-measure=\frac{2tp}{2tp+fp+fn}$$

(4) $$Accuracy=\frac{tp+tn}{tp+tn+fp+fn}$$

True positives (tp) represent successfully selected gene markers and false positives (fp) as incorrect selected gene markers. False negatives (fn) represent incorrectly discarded gene markers and true negatives (tn) as correctly discarded non-gene markers. Table 2 summarizes precision, recall, F-measure, and accuracy of the combined filters. In terms of precision, with no filtering method, there was only 0.002 of chance to select a gene marker and 0.011 for the DEG-only filtering method (Table 2). Both non-filtered and DEG-only filtered methods were not powerful enough to detect potential gene markers in terms of the performance measures above. However, when we gradually added GRN, pathway, and SNV filters, the performance improved dramatically. In terms of precision, GRN pathway, and SNV filters achieved 0.041. About 4 times higher precision compared to the DEG-only filter. Naturally higher precision comes with lower recall rates. However, reduction in recall was not as dramatic as precision, reporting 0.538. When we consider both precision and recall, it was evident that our filtering method outperforms the DEG-only method in terms of both F-measure and accuracy. For the F-measure, GRN, pathway, and SNV filters achieved 0.076 that is 3.6 times higher F-measure than the DEG-only filter. For accuracy, GRN, pathway, and SNV filters achieved 0.972 that is 1.2 times higher accuracy when compared to the DEG-only filter. In summary, our proposing method, the combination analysis of DEG, GRN, pathway, and SNV filters out performs the DEG-only filtering method in terms of predicting potential gene markers.

**Table 2:** Performance comparison of gene marker prediction methods.

Gene Symbol	NONE	DEG	DEG+GRN	DEG+GRN+Pathway	DEG+GRN+Pathway+SNV
Relb	HIT	HIT	HIT	HIT	HIT
Nfkb2	HIT	HIT	HIT	HIT	HIT
Tnfrsf9	HIT	HIT	HIT	HIT	HIT
Tnfrsf21					
Icos	HIT	HIT	HIT	HIT	HIT
Il2ra	HIT	HIT	HIT	HIT	HIT
Cysltr1	HIT	HIT			
Kit	HIT	HIT	HIT	HIT	HIT
Il1r2	HIT	HIT	HIT	HIT	
Il13	HIT	HIT	HIT	HIT	HIT
Il5	HIT	HIT	HIT	HIT	HIT
Areg	HIT	HIT	HIT	HIT	HIT
Il1rl1	HIT	HIT	HIT		
Ccr8	HIT	HIT			
Tph1	HIT	HIT			
Htr1b					
Cd244	HIT	HIT	HIT	HIT	
Lta	HIT	HIT	HIT	HIT	
Il10	HIT	HIT	HIT	HIT	HIT
Tnf	HIT	HIT	HIT	HIT	HIT
Nfkbia	HIT	HIT	HIT	HIT	
Cdkn2b	HIT	HIT	HIT	HIT	HIT
Lif	HIT	HIT	HIT	HIT	HIT
Il2ra	HIT	HIT	HIT	HIT	HIT
Il9r	HIT	HIT			
Il24					

Num of Hits	23	23	19	18	14
Selected Candidates	12298	2153	1184	478	343

Precision	0.002	0.011	0.016	0.038	0.041
Recall	0.885	0.885	0.731	0.692	0.538
F-measure	0.004	0.021	0.032	0.073	0.076
Accuracy	0.002	0.827	0.905	0.962	0.972

## Conclusion

We proposed a novel method that use a four-step fil-tering strategy in order to find potential gene markers from RNA-seq data of mouse knockout studies that have small number of samples. The standard DEG method was used to select candidates that explains the phenotypic differences between KO and WT in the first filtering step. A combination of GRN, biological pathways and SNV information was able to narrow down significant genes that have regulatory role and reduced the risk of including candidates that have genetic differences. As a result, we were able to distinguish multiple gene markers in a small number of samples by reproducing the research findings reported in a knockout study [12]. Moreover, the use of KEGG pathways [10] for gene marker selection can be viewed as utilizing the prior information since pathways are constructed based on information from the literature. Thus, mapping potential gene markers to the pathways takes advantage of the interpretability of omics data from mouse knockout studies.

However, several limitations of this study need to be addressed. First of all, there should be more rigorous study of GRN construction. Using much omics data for GRN construction somehow preserves important relationships between transcription factors and their target genes, but how much data is needed for GRN construction is not rigorously studied. In this study, we had enough omics data for the network construction, therefore we were able to use a simple method using NARROMI[9]. However, when the amount of omics data for network construction is not enough, special techniques such as low order partial correlation based methods [13] should be considered. Second, removing genes with genetic variation allows us to focus on genes that are relevant to the underlying biological mechanisms for the knockout study. How-ever, genetic variations do not necessarily affect transcription activity of genes, and it is possible that our method might discard some SNVs that were affected by the knockout gene. Thus we need to further investigate the effect of genetic variations on transcription activities. Finally, we plan to develop a machine learning based gene ranking method so that genes selected at the last step can be ranked and help biologists to prioritize the follow up study after the knockout study.

## Methods

### Data Collection

Public data (Microarray, RNA-seq) of mice were collected from NCBI GEO. For Microarray, each series matrix files from GSE45929 [14], GSE16741 [15], GSE30906 [16], GSE36780 [17], GSE40375 (not published), GSE41380 [18], GSE43663 [19] were used for GRN construction. These data contains gene expression value of multiple samples (42 samples in total) that were created by the same microarray platform (Illumina MouseWG-6 v2.0 expression beadchip) and preprocessed by R bioconductor lumi package [20] (variance stabilizing transform, quantile normalization). Note that these samples differs in mouse's strain, genotype and treatment. For RNA-seq data, GSE47851 were used for the evaluation of the study's method. RNA-seq data of GSE47851 are from an experiment of Gata3 KO that have multiple SRA files. We used 8 SRA files (SRR896215, SRR896216, SRR896217, SRR896218, SRR896219, SRR896220, SRR896221, SRR896222) that are raw data in two conditions where each of the conditions have 2 biological samples and 2 technical replicates of each biological sample.

### Gene regulatory network construction

We integrated gene expression values of 7 series matrix files (GSE45929, GSE16741, GSE30906, GSE36780, GSE40375, GSE41380, GSE43663) into a single matrix and quantile normalized gene expression values of every sample and used it as an expression profile for construction of GRN. GRN is constructed by using NARROMI [9] with a default option. As NARROMI requires a list of transcription factors and a list of genes, we collected a list of transcription factors and co-factors from the Animal Transcription Factor Database [21] and defined this as a transcription factor list. For the gene list, we simply defined it as a list of whole genes that includes not only transcription factors and co-factors but also non-transcription factors. As a result NARROMI constructed a network topology of 2950865 edges. We support a URL for the network topology file which was used in this study. (http://epigenomics.snu.ac.kr/mouse_network/total_mouse.topology)

### Preprocess of RNA-seq

An annotation file and reference genome from ENSEMBL (Mus musculus.GRCm38.70) [22] were used. Samples that have technical replicates were integrated into a single file and trimmed by using Trim Galore [23]. Trimmed sequences were then aligned by using Tophat [24] with '-G' option. After sequences were aligned, we used Cuffdiff [25] by inputting biological replicates with a default option. Genes that have FPKM under 1 are excluded, since these genes tend to have high fold changes and most of them are artifacts of low gene expression values.

### SNV Calling

Samtools mpileup [26] was used for SNV calling. We used accepted hits.bam which was created by Tophat results. Options used in Samtools were 'mpileup --uf' and 'view -bvcg' for bcftools. Results of Sam-tools were filtered by varFilter with '-D100' option. As a result, 4 vcf files (WT1, WT2, KO1, KO2) were produced. In order to observe SNV differences between WT and KO, we discarded insertions and deletions from each vcf file. Then we counted the number of mutations of each gene in each vcf file and calculated the average of mutation in WT and KO. Finally, we compared the rates of mutation for each gene between WT and KO, mutation rate (fold change) greater than 2 were considered as possible genetic variation effects.

## Competing interests

The authors declare that they have no competing interests.

## Authors' contributions

SK designed and supervised the research project and edited the paper. HC contributed in SNV analysis and advised helpful information in preprocess of RNA-seq. Other data process, data analysis, paper work was done by BH.
